# MicroRNA‐107 regulates anesthesia‐induced neural injury in embryonic stem cell derived neurons

**DOI:** 10.1002/iub.1911

**Published:** 2018-10-11

**Authors:** Jun Dan Jiang, Xiao Chun Zheng, Feng Yi Huang, Fei Gao, Mei Zhen You, Ting Zheng

**Affiliations:** ^1^ Department of Anesthesiology Fujian Provincial Hospital, Provincial Clinical Medical College, Fujian Medical University Fujian China; ^2^ Department of Anesthesiology Fujian Provincial Emergency Center, Provincial Clinical Medical College, Fujian Medical University Fujian China

**Keywords:** BDNF, embryonic stem cell, ketamine, microRNA, miR‐107, neural injury

## Abstract

Ketamine, though widely used in pediatric anesthesia, may induce cortical neurotoxicity in young patients. This study focused on an *in vitro* model of rat brain embryonic stem cell (ESC)‐derived neurons to investigate the effects of microRNA‐107 (miR‐107) on ketamine‐induced neural injury. Rat brain ESCs were proliferated *in vitro* and differentiated toward neuronal fate. Ketamine induced neural injury in ESC‐derived neurons was inspected by TUNEL and neurite growth assays. Ketamine‐induce aberrant miR‐107 expression was examined by qRT‐PCR. MiR‐107 was downregulated in ESCs through lentiviral transduction. Its effect on ketamine‐induced neural injury in ESC‐derived neurons was then examined. Potential downstream target of miR‐107, brain derived neurotrophin factor (BDNF), was inspected by dual‐luciferase reporter assay and qRT‐PCR. BDNF was knocked down, through siRNA transfection, in NSCs to investigate its functional involvement in miR‐107 mediated neural protection in ketamine‐injured NSC‐derived neurons. Ketamine induced apoptosis, neurite degeneration, and upregulated miR‐107 in NSC‐derived neurons. Lentivirus‐mediated miR‐107 downregulation attenuated ketamine‐induced neural injury. BDNF was proven to be directly and inversely regulated by miR‐107 in NSC‐derived neurons. SiRNA‐mediated BDNF inhibition reversed the protective effect of miR‐107 downregulation on ketamine injury in NSC‐derived neurons. MiR‐107 / BDNF was demonstrated to be an important epigenetic signaling pathway in regulating ketamine‐induced neural injury in cortical neurons. © 2018 The Authors. IUBMB Life published by Wiley Periodicals,Inc. on behalf of International Union of Biochemistry and Molecular Biology., 71(1):20–27, 2019

**Abbreviations**
*3’‐UTR*: *3′‐untranslated region*
*BDNF*: *brain derived neurotrophin factor*
*ESC*: *embryonic stem cell*
*miRNA*: *microRNA*
*NMDA*: *N‐methyl‐D‐aspartate*

## INTRODUCTION

Ketamine, specified as an N‐methyl‐D‐aspartate (NMDA) receptor blocker, is broadly used in pediatric anesthesia 1, 2. Yet, studies in recent decades demonstrated that ketamine might induce cortical neural injury, leading to apoptosis, neurite degeneration, or even cell death in neonatal or young brains in both animal models and human patients 3, 4, 5, 6, 7. The underlying molecular mechanisms contributing to ketamine‐induced cortical neural injury may be complicated, as various singling pathways were suggested to be actively involved in this neurotoxic process. For instance, 7‐nitroindazole, a nitric oxide synthase inhibitor, was shown to protect ketamine‐induced neurotoxicity in rat forebrain by reducing polysialic acid neural cell adhesion molecule (PSA‐NCAM) and Bax/BCL‐XL ratio 8. In addition, Dexmedetomidine, a potent α2‐adrenoceptor agonist, ameliorated hippocampal CA1 neuron apoptosis and improved learning and memory capabilities in ketamine‐injured rats 9.

MicroRNAs (miRNAs) are families of small (18–22 n.t. long), noncoding RNAs that attach to the complementary binding site on 3′‐untranslated region (3’‐UTR) of downstream target genes, thus post‐transcriptionally inhibiting gene and protein productions 10, 11, 12. Through such genetic control mechanism, miRNAs have been shown to be actively involved in almost all aspects of biological regulations in plants, animals and human beings 13, 14, 15, 16, 17, 18. Recently, several miRNA candidates, such as miR‐206, miR‐124, and miR‐34a had been identified as potential epigamic regulators to modulated ketamine‐induced neurotoxicity in hippocampal neurons 19, 20, 21.

Among many of the brain‐associated miRNAs, microRNA‐107 (miR‐107) has been shown to be correlated with several neurodegenerative diseases. For instance, microarray study on human brain tissues revealed that miR‐107 was aberrantly downregulated in patients with Alzheimer's disease, even in early stages 22. Also, a very recent study demonstrated that in animal model of Alzheimer's disease, reduced miR‐107 level was closely associated with memory impairment and cortical neuronal loss, whereas increased miR‐107 reversed such process 23. In addition, in a study focusing on focal cerebral ischaemia/reperfusion (I/R)‐induce brain injury, miR‐107 was found to be significantly upregulated in both brain tissues and plasma samples 24. Conversely, utilizing miR‐107 inhibitor effectively rescued I/R‐induced cortical neurotoxicity 24.

Recently, *in vitro* models of stem cell derived neurons had been developed to investigate the molecular mechanisms of anesthesia‐induced neurotoxicity 25, 26, 27. Thus, in the present study, we took advantage of an *in vitro* animal model 27, to investigate the expression and function of miR‐107 in ketamine‐induced neural injury in rat brain embryonic neural stem cell (NSC)‐derived neurons.

## MATERIALS AND METHODS

### Ethics and Consent

In this study, all animal protocols were approved by the Clinical Research and Ethics Committee at Fujian Provincial Hospital, Provincial Clinical Medical College and Fujian Medical University in Fuzhou, Fujian Province in China. All procedures were conducted according to the 8th edition of National Institute of Health Guide for the Care and Use of Laboratory Animals 28.

### Rat Embryonic Stem Cells Derived Neurons and Ketamine Treatment

*In vitro* neuronal induction of rat brain embryonic stem cells (ESCs) was conducted according to the methods described before but with slight variations 25, 27, 29. Briefly, pregnant Sprague–Dawley rats, at embryonic/gestational day 16.5–18.5, were euthanized and sacrificed. Rat embryonic brains were quickly retrieved and mechanically dissociated into a 35 cm glass dish containing ice‐cold Hanks’ balanced salt solution without Calcium, Magnesium or Phenol Red (HBSS, ThermoFisher Scientific, USA). After centrifuged at 150 × g for 15 mins at 4 °C, pellets were resuspended in ESC neural proliferation medium, which was DMEM/F12 with HEPES & L‐Glutamine (ThermoFisher Scientific, USA) supplemented with N‐2 supplement (1X, ThermoFisher Scientific, USA) and EGF/bFGF (10 ng/mL, ThermoFisher Scientific, USA), in a 6‐well plate (Greiner, Germany) at 37 °C in 5% CO_2_. After 3–5 passages, neural ESCs were treated with transition medium, which was DMEM/F12 supplemented with N‐2, EGF/bFGF/PDGF, and 5% fetal bovine serum (FBS, ThermoFisher Scientific, USA) for 24 h, followed by exposure to differentiation medium, which was DMEM/F12 supplemented with N‐2 and 10% FBS for 1 week. Then, ESC‐derived neurons were treated with ketamine (MilliporeSigma, Shanghai, China) at various concentrations for 24 h. After that, ketamine was removed and ESC‐derived neurons were incubated with differentiation medium for 2 or 7 days, prior to further processings.

### Apoptosis Assay

Apoptosis was measured in ESC‐derived neurons according to the methods described before but with slight variations 27, 29, 30. Briefly, an Alexa Fluo‐594 dye‐conjugated Click‐iT™ Plus TUNEL Assay (ThermoFisher Scientific, USA) was used, according to the manufacturer's recommendations, to detect in situ apoptosis among ESC‐derived neurons. Also, an Alexa Fluor‐488‐conjudated rabbit monoclonal NeuN antibody (Neuronal Marker, Abcam, USA) was used to detect ESC‐derived neurons. Processed 6‐well plates were then mounted an inverted fluorescent microscopy system and examined under 20× objective with TRTIC / FTIC filters (Axio Observer A1, Zeiss, Germany). Neuronal apoptosis was then characterized as the averaged percentage of TUNEL+ cells among TUNEL+ & NeuN+ cells.

### Neurite Growth Assay

Neurite growth was measured in ESC‐derived neurons according to the methods described before but with slight variations 29, 30. Briefly, ESC‐derived neurons were quickly fixed by 4% paraformaldehyde (PFA, ThermoFisher Scientific, USA) in phosphate‐buffer solution (PBS, ThermoFisher Scientific, USA) for 20  min, and incubated with a blocking medium, which was PBS containing 5% normal horse serum (ThermoFisher Scientific, USA) and 0.1% Triton (MilliporeSigma, Shanghai, China) for 1 hour. To detect neurites of ESC‐derived neurons, a mouse monoclonal Tuj‐1 primary antibody (Santa Cruz, USA) was applied for 24  h at 4 °C, followed by a goat‐antimouse Alexa Fluor‐594 secondary antibody (ThermoFisher Scientific, USA) for 2  h at room temperature. The Alexa Fluor‐488‐conjudated rabbit monoclonal NeuN antibody was also applied to detect ESC‐derived neurons, specifically. Processed 6‐well plates were then mounted the inverted fluorescent microscopy system and examined under 20× objective with TRTIC/FTIC filters (Axio Observer A1, Zeiss, Germany). In each well, 3 areas (250 X 250 μM) were randomly selected and the averaged TRITC fluorescent intensity was measured. Relative neuronal outgrowth was then characterized by normalizing those averages against the average under control condition.

### RNA Extraction and Quantitative Real‐Time PCR (qRT‐PCR)

Total RNA was extracted from ESC‐derived neurons using a Tri‐reagent Kit (Molecular Research Center, USA). The reverse transcription was conducted using an AccuPower RT‐Premix kit (Bioneer, Republic of Korea). The quantitative real‐time polymerase chain reaction (qRT‐PCR) was conducted on an ABI Prism 7000 real‐time PCR system (Applied Biosystems, CA). For detection of miR‐107, a *mir*Vana™ qRT‐PCR miRNA Detection Kit (Applied Biosystems, USA) was used according to the manufacturer's recommendations. For detection of BDNF, a SYBR Green qRT‐PCR kit (Applied Biosystems, USA) was alternatively used according to the manufacturer's recommendations. Relative gene expression level was then calculated as fold changes using the 2^(−ΔΔCt)^ method.

### Lentivirus Assay

A chemically synthetized rno‐miR‐107 inhibitor lentivirus (Lenti‐107/I), and a rat control miRNA inhibitor lentivirus (lenti‐C) were commercially obtained from RiboBio (RiboBio, Guangzhou, China). NSCs were transduced with Lenti‐107/I or Lenti‐C, in the presence of polybrene (8 μg/mL, MilliporeSigma, Shanghai, China) for 48 h at multiplicity of infection (MOI) of 25, followed by blasticidin selection (8 μg/mL, MilliporeSigma, Shanghai, China) for 3 days. Healthy NSC colonies were collected and re‐plated in NSC proliferation medium. After 3rd passage, NSCs were exposed to transition medium for 1 day and then neural differentiation medium for 1 week. QRT‐PCR was conducted to examine the efficiency of miR‐107 downregulation in lentivirus‐transduced NSC‐derived neurons.

### Dual‐Luciferase Reporter Assay

A chemically synthetized rno‐miR‐107 mimics (RmiR‐107‐m), and a rat control miRNA mimics (RmiR‐C) were commercially obtained from RiboBio (RiboBio, Guangzhou, China). In addition, a pmiR‐REPORT luciferase plasmids containing a wild type (wt) rat BDNF 3’‐UTR (luc_BDNF_wt), and a mutated (mu, at putative rno‐miR‐107 binding site) rat BDNF 3’‐UTR (luc_BDNF_mu) were also commercially obtained from RiboBio (RiboBio, Guangzhou, China). Human HEK293T cells were cotransfected with RmiR‐107‐m (or RmiR‐C) and luc_BDNF_wt (or luc_BDNF_mu) for 48 h. A Dual‐Luciferase Reporter Assay (Promega, USA) was then conducted according to the manufacturer's recommendations.

### SiRNA Assay

A chemically synthetized rat BDNF‐specific siRNA (Si‐RBDNF), and a scrambled control siRNA (si‐C) were commercially obtained from RiboBio (RiboBio, Guangzhou, China). Lenti‐107/I‐transduced NSCs were transfected with siRNAs for 24  h, exposed to transition medium for 1 day and then differentiation medium for 1 week. QRT‐PCR was then conducted to examine the efficiency of BDNF downregulation in lentivirus‐transduced and siRNA‐transfected NSC‐derived neurons.

### Statistical Analysis

In this study, all assays were repeated for at least three times. Summarized data were presented as mean  ±standard errors. Statistical significance was compared between summarized data using Student *t*‐test. *P*  <  0.05 indicates significant difference.

## RESULTS

### Ketamine Induced Apoptosis, Neurite Degeneration and miR‐107 Upregulation in Embryonic Stem Cells‐Derived Neurons

**Figure 1:**
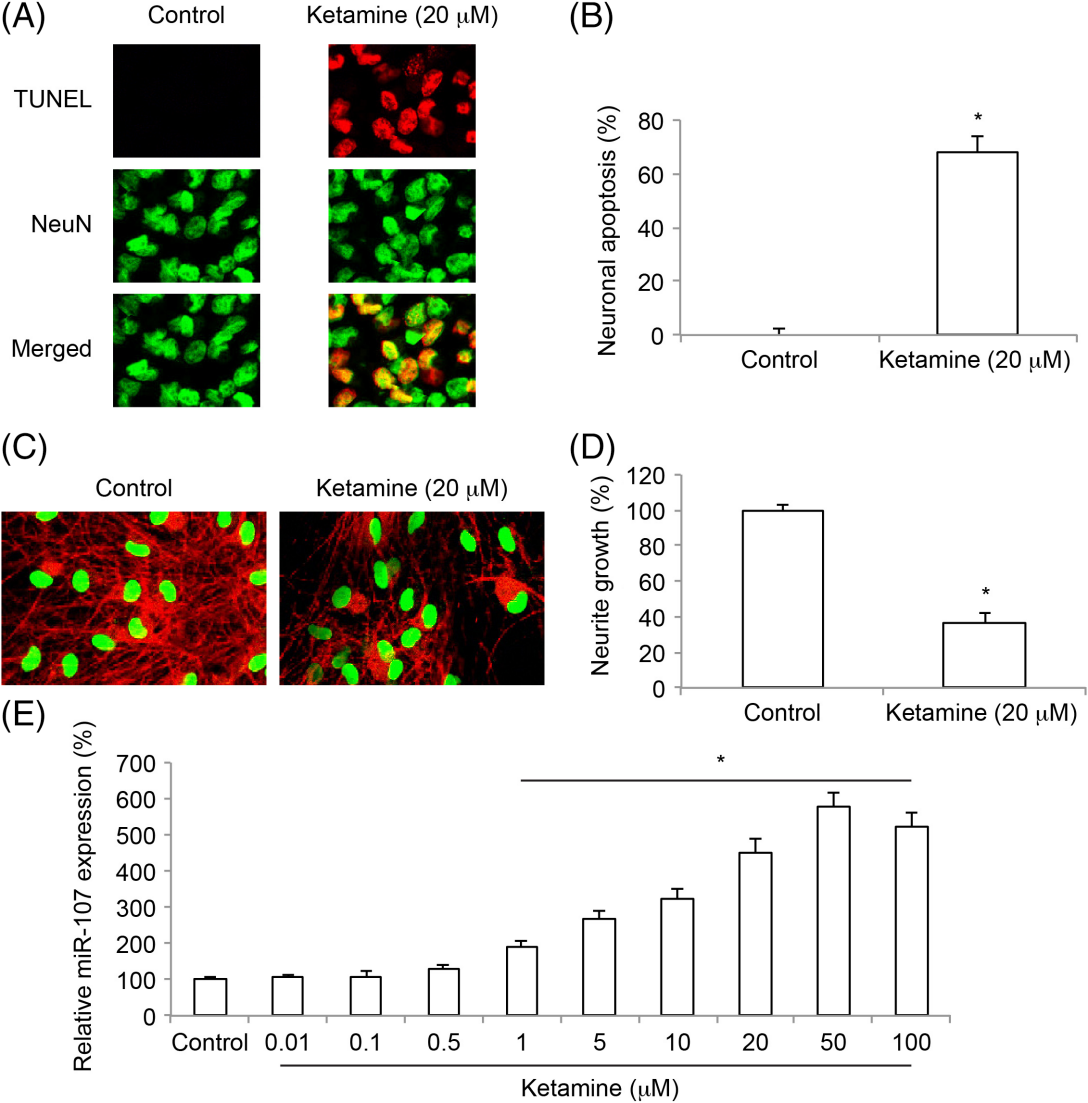
Ketamine‐induced neuro‐injury and aberrant miR‐107 expression in ESC‐derived neurons. (A) Rat brain embryonic stem cells (ESCs) were proliferated *in vitro*. ESCs were differentiated toward neuronal fate for 7 days, before they were exposed to 20 μM (or 0 μM, Control) ketamine for 24 h. After that, ketamine was removed and ESC‐derived neurons were kept in differentiation medium for additional 2 days. Then, a TUNEL assay (Red), along with a fluorescence‐conjugated NeuN neuronal marker antibody (Green) was applied. Representative fluorescent images were shown for ESC‐derived neurons under control condition and neurons exposed to 20 μM ketamine. (B) For (A), neuronal apoptosis was quantified between ESC‐derived neurons under control condition and neurons exposed to 20 μM ketamine (*P < 0.05). (C) After ketamine was removed, ESC‐derived neurons were kept in differentiation medium for additional 7 days. Then, immunohistochemistry using Tuj‐1 (Red) and NeuN (Green) antibodies was conducted. Representative fluorescent images were shown for ESC‐derived neurons under control condition and neurons exposed to 20 μM ketamine. (D) For (C), relative neurite growth was quantified between ESC‐derived neurons under control condition and neurons exposed to 20 μM ketamine (*P < 0.05). (E) ESC‐derived neurons were also exposed to various concentrations of ketamine (0–100 μM) for 24 h. Then, 2 days after ketamine was removed, qRT‐PCR was conducted on ESC‐derived neurons to examine miR‐107 expression in response to ketamine exposure (* *P* < 0.05).

Rat brain ESCs were cultured *in vitro* and differentiated toward neuronal fate. ESC‐derived neurons were exposed to ketamine (20 μM) for 24 h. Forty‐eight hours after ketamine was removed, a TUNEL assay was conducted. Imaging examination demonstrated that, as compared to the condition without ketamine exposure (Control), considerable TUNEL immunostaining was seen in ketamine‐exposed ESC‐derived neurons (Fig. 1A). Then, quantitative measurement on neuronal apoptosis confirmed that, ketamine induced significant apoptosis in ESC‐derived neurons (Fig. 1B, *P < 0.05). Seven days after ketamine exposure, immunohistochemistry of Tuj‐1 (Red) and NeuN (Green) staining was conducted. It showed that, as compared to the Control condition, considerably less Tuj‐1‐positive neurites were seen in ketamine‐exposed derived neurons (Fig. 1C). Moreover, quantitative measurement on neuronal growth confirmed that, ketamine induced significant neurite degeneration in ESC‐derived neurons (Fig. 1D, *P < 0.05). ESC‐derived neurons were also exposed to ketamine at various concentrations (0– 100 μM) for 24 h. Two days after ketamine was removed we measured miR‐107 expression. The results of qRT‐PCR demonstrated that miR‐107 was upregulated by ketamine in concentration‐dependent manner in ESC‐derived neurons (Fig. 1E, *P < 0.05).

### MiR‐107 Downregulation Attenuated Ketamine‐Induced Neural Injury in Embryonic Stem Cells‐Derived Neurons

**Figure 2:**
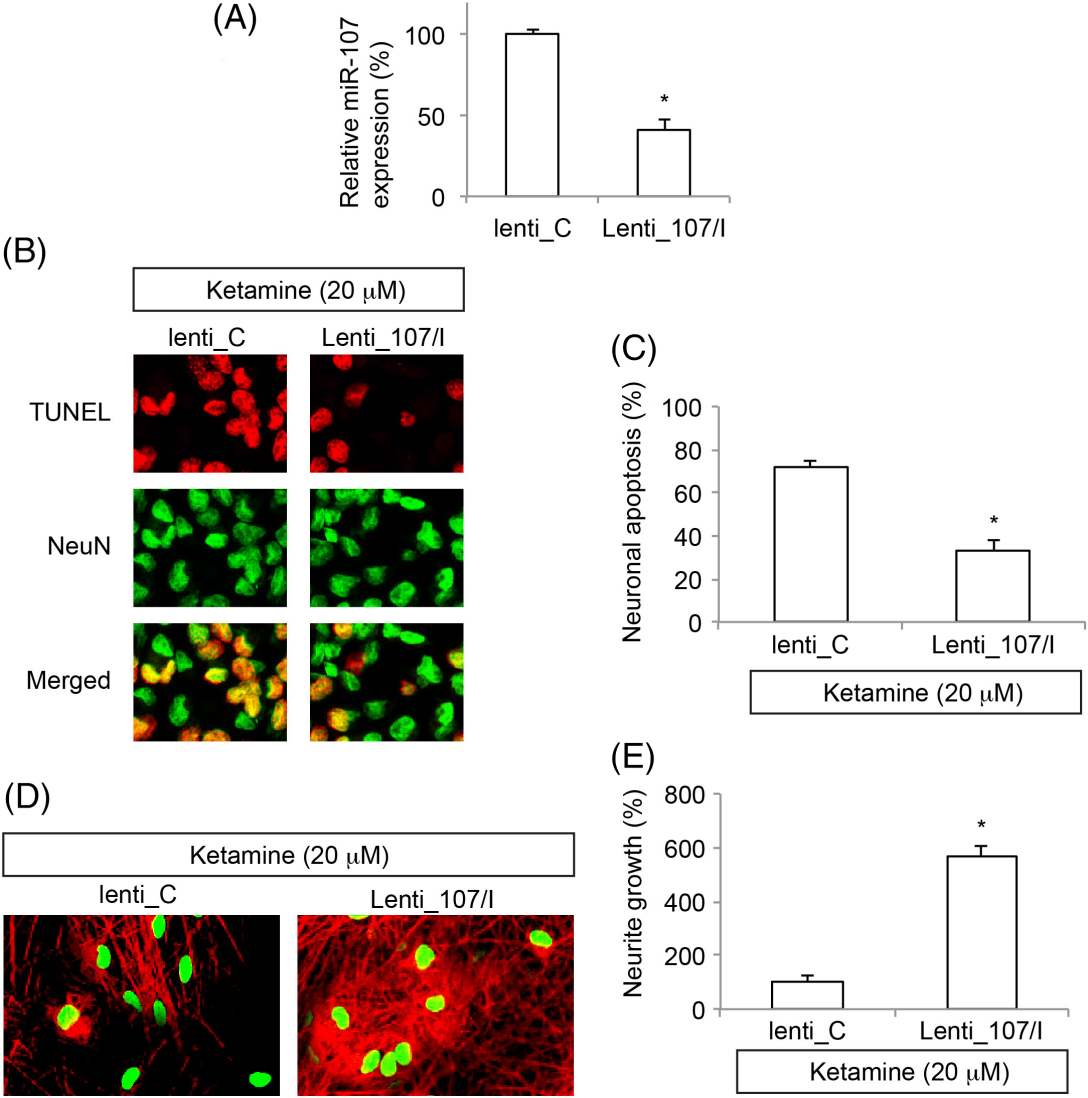
MiR‐107 downregulation‐mediated neural protection in ketamine‐injured ESC‐derived neurons. (A) ESCs were transduced with a chemically synthetized rno‐miR‐107 inhibitor lentivirus (Lenti‐107/I), or a rat control miRNA inhibitor lentivirus (lenti‐C). After transduction was stabilized, ESCs were induced toward neuronal differentiation. After 7 days in differentiation medium, qRT‐PCR was conducted to compare miR‐107 expressions between lenti‐C‐ and Lenti‐107/I‐ transduced NSC‐derived neurons (*P < 0.05). (B) ESC‐derived neurons were exposed to 20 μM ketamine for 24 h. After that ketamine was removed and ESC‐derived neurons were kept in differentiation medium for additional 2 days. Then, a TUNEL assay was applied. Representative fluorescent images were shown for lenti‐C‐ and Lenti‐107/I‐ transduced ESC‐derived neurons. (C) For (B), neuronal apoptosis was quantified (* *P* < 0.05). (D) After ketamine was removed, lentivirus‐transduced ESC‐derived neurons were kept in differentiation medium for additional 7 days, followed by immunohistochemistry using Tuj‐1 (Red) and NeuN (Green) antibodies. Representative fluorescent images were shown for lenti‐C‐ and Lenti‐107/I‐ transduced ESC‐derived neurons. (E) For (D), relative neurite growth was quantified (*P < 0.05).

As we discovered that miR‐107 was upregulated by ketamine in ESC‐derived neurons, we wondered whether miR‐107 might functionally regulate ketamine‐induced neural injury. To explore this hypothesis, ESCs were transduced with miR‐107 inhibitor lentivirus (Lenti‐107/I) or a control inhibitor lentivirus (lenti‐C), and then differentiated toward neuronal fate. After exposed to differentiation medium for 7 days, qRT‐PCR showed endogenous miR‐107 expression was significantly downregulated in ESC‐derived neurons transduced with Lenti‐107/I than in neurons transduced with lenti‐C (Fig. 2A, *P < 0.05)

Lentivirus‐transduced neurons were then exposed to 20 μM ketamine for 24 h. Forty‐eight hour after ketamine was removed, the imaging result of TUNEL assay showed that, considerably less TUNEL staining was seen in NSC‐derived neurons transduced with Lenti‐107/I than in neurons transduced with lenti‐C (Fig. 2B). Then, quantification on neuronal apoptosis confirmed that miR‐107 downregulation significantly attenuated ketamine‐induced apoptosis in NSC‐derived neurons (Fig. 2C, *P < 0.05). Moreover, 7 days after ketamine was removed, immunostaining of Tuj‐1 (Red) and NeuN (Green) antibodies showed that considerably more Tuj‐1‐positive neurites were seen in NSC‐derived neurons transduced with Lenti‐107/I than in neurons transduced with lenti‐C (Fig. 2D). Quantification on relative neurite growth confirmed our observation, showing that miR‐107 downregulation significantly prevented ketamine‐induced neurite degeneration in NSC‐derived neurons (Fig. 2E, *P < 0.05).

### BDNF was Inversely Correlated with miR‐107 in Ketamine‐Injured Embryonic Stem Cells‐Derived Neurons

**Figure 3:**
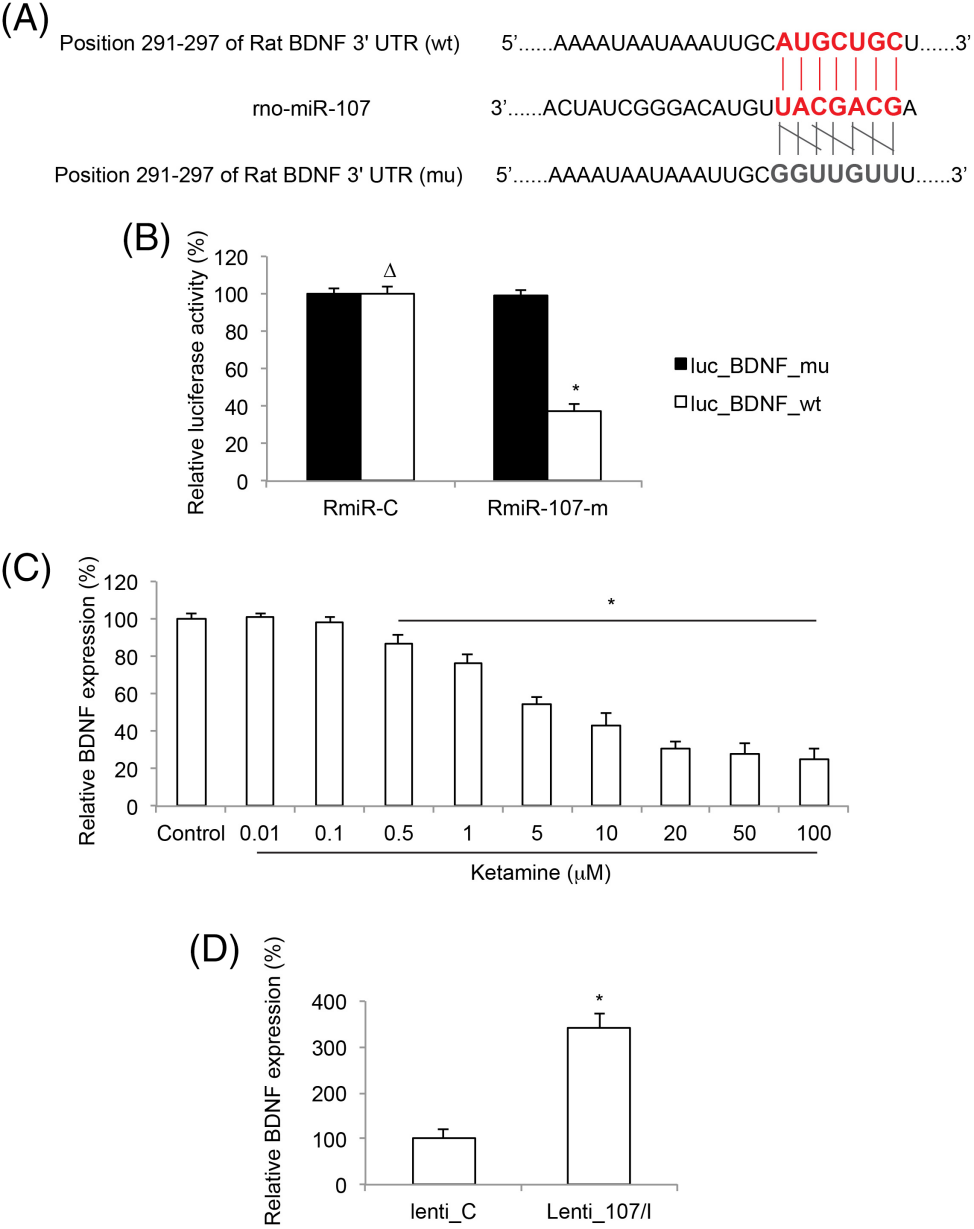
Ketamine and miR‐107 mediated BDNF expression in ESC‐derived neurons. (A) A carton was demonstrated for 3’‐UTR of wild type (wt) rat BDNF gene, which includes a putative rno‐miR‐107 binding sequence. A mutated (mu) rat BDNF 3’‐UTR was correspondingly constructed to void rno‐miR‐107 binding. (B) In a dual‐luciferase reporter assay, HEK293T cells were double transfected with RmiR‐107‐m or RmiR‐C, and luc_BDNF_wt or luc_BDNF_mu. Two days later, relative luciferase activities were measured (* *P* < 0.05; Δ *P* > 0.05). (C) After incubated in differentiation medium for 7 days, ESC‐derived neurons were exposed to various concentrations of ketamine (0–100 μM) for 24 h. Two days after ketamine was removed, qRT‐PCR was conducted on ESC‐derived neurons to examine BDNF gene expression in response to ketamine exposure (* *P* < 0.05). (D) Lentivirus‐transduced ESCs were differentiated toward neurons. After exposure to differentiation medium for 7 days, qRT‐PCR was conducted to compare BDNF expressions in lenti‐C‐ and Lenti‐107/I‐transduced ESC‐derived neurons (* *P* < 0.05).

Using miRNA targeting algorithm (www.targetscan.org), we noticed that BDNF was likely one of the downstream target genes of miR‐107 in rat (Fig. 3A). In a dual‐luciferase reporter assay, we double‐transfected HEK293T cells with rat miR‐107 mimics (RmiR‐107‐m) or a rat control miRNA mimics (RmiR‐C), along with a luciferase vector containing wild type rat BDNF 3′‐UTR (luc_BDNF_wt, Fig. 3A) or a luciferase vector containing a mutated rat BDNF 3′‐UTR without rno‐miR‐107 binding site (luc_BDNF_mu, Fig. 3A). The measurement on relative luciferase activity confirmed that rat BDNF was the direct downstream target of rno‐miR‐107 (Fig. 3B, *P < 0.05, Δ *P* > 0.05).

We then measured BDNF gene expression in NSC‐derived neurons after they were exposed to different concentrations of ketamine. The result of qRT‐PCR showed that BDNF was downregulated by ketamine in concentration‐dependent manner, opposite to the upregulated pattern of miR‐107 in ESC‐derived neurons (Fig. 3C, *P < 0.05). We also measured BDNF gene expression in lentivirus‐transduced ESC‐derived neurons. QRT‐PCR demonstrated that, BDNF was inversely upregulated in Lenti‐107/I‐transduced neurons, than in lenti‐C‐transduced neurons (Fig. 3D, *P < 0.05).

### BDNF was Functionally Involved in miR‐107‐Mediated Protection on Ketamine‐Induced Neural Injury in Embryonic Stem Cells‐Derived Neurons

**Figure 4:**
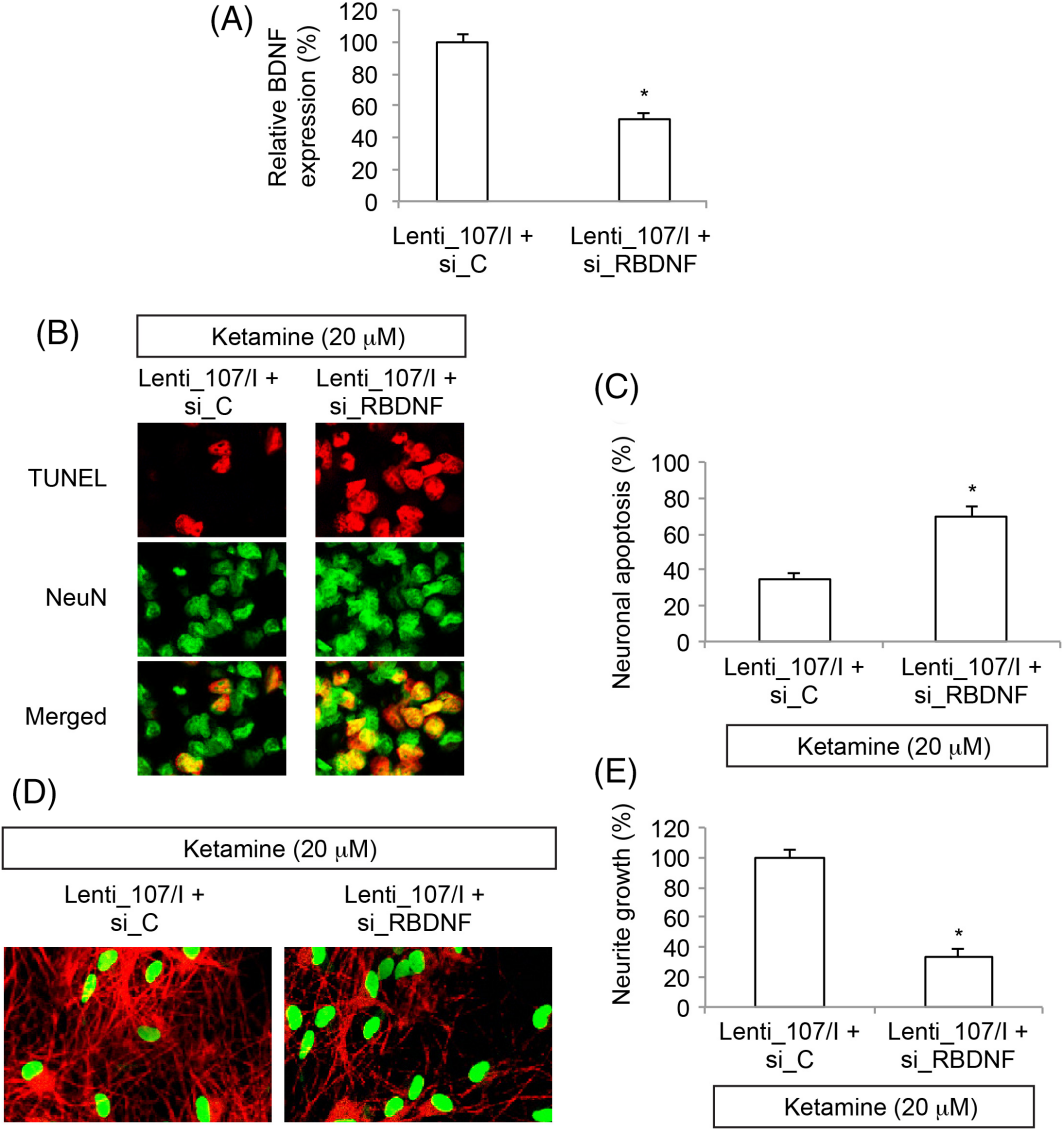
BDNF inhibition reversed the protective effect of miR‐107 downregulation on ketamine‐induced neural injury in ESC‐derived neurons**.** (A) Lenti‐107/I‐transduced ESCs were transfected with si_C or si_RBDNF, then differentiated toward neuronal fate. After 7 days in differentiation medium, qRT‐PCR was conducted to compare BDNF expressions between si_C‐ and si_RBDNF‐ transfected NSC‐derived neurons (* *P* < 0.05). (B) ESC‐derived neurons were exposed to 20 μM ketamine for 24 h. After that ketamine was removed and ESC‐derived neurons were kept in differentiation medium for additional 2 days. Then, a TUNEL assay was applied. Representative fluorescent images were shown for Lenti‐107/I‐transduced ESC‐derived neurons, which were transfected with si_C or si_RBDNF. (C) For (B), neuronal apoptosis was quantified (* *P* < 0.05). (D) After ketamine was removed, lentivirus‐transduced ESC‐derived neurons were kept in differentiation medium for additional 7 days, followed by immunohistochemistry using Tuj‐1 (Red) and NeuN (Green) antibodies. Representative fluorescent images were shown for Lenti‐107/I‐transduced ESC‐derived neurons, which were transfected with si_C or si_RBDNF. (E) For (D), relative neurite growth was quantified (*P < 0.05).

Finally, we examined whether miR‐107‐inhibiton‐mediated protection on ketamine‐induced neural injury may act through BDNF. In NSCs transduced with Lenti‐107/I, we further transfected them with a rat BDNF‐specific siRNA (Si‐RBDNF), or a scrambled control siRNA (si‐C) for 24 h. NSCs were then differentiated toward neuronal fate. After exposed to differentiation medium for 7 days, qRT‐PCR showed endogenous BDNF was significantly downregulated in Lenti‐107/I‐transduced ESC‐derived neurons that were transfected with si_RBDNF, than in neurons transfected with si_C (Fig. 4A, *P < 0.05).

These NSC‐derived neurons were exposed to 20 μM Ketamine. Forty‐eight hour after ketamine was removed, the imaging result of TUNEL assay showed that, in Lenti‐107/I‐transduced NSC‐derived neurons, considerably more TUNEL staining was seen in neurons transfected with si_RBDNF than in neurons transfected with si_C (Fig. 4B). Quantification on neuronal apoptosis confirmed that BDNF inhibition reversed miR‐107 downregulation mediated protection on ketamine‐induced apoptosis in NSC‐derived neurons (Fig. 4C, *P < 0.05). Furthermore, 7 days after ketamine was removed, immunostaining of Tuj‐1 (Red) and NeuN (Green) antibodies showed that, in Lenti‐107/I‐transduced NSC‐derived neurons, significantly less Tuj‐1‐positive neurites were seen in neurons transfected with si_RBDNF than in neurons transfected with si_C (Fig. 4D). Then, quantification on relative neurite growth confirmed that BDNF inhibition significantly reduced the protective effect of miR‐107 downregulation on ketamine‐induced neurite degeneration in NSC‐derived neurons (Fig. 4E, *P < 0.05).

## DISCUSSIONS

Recent studies demonstrated that epigenetic regulators, such as long noncoding RNAs, might modulate anesthesia‐induced neurotoxicity in neural stem cells‐derived neurons 29, 31. In this study, we used an *in vitro* model of rat brain ESC‐derived neurons to demonstrate that, another category of epigenetic regulators, microRNA, may play a critical role in regulating ketamine‐induced neural injury in cortical neurons.

Firstly, we applied a known neuronal differentiation and ketamine neurotoxicity protocol 27, to induce neural injury in rat brain NSC‐derived neurons. We demonstrated that, exposure to 20 μM ketamine caused significant apoptosis and neurite degeneration in NSC‐derived neurons. This result was consistent with a previous study showing similar neurotoxic effect of ketamine in neural stem cell‐derived rat cortical neurons 29. However, there was a major difference between our study and that study, which was that growth factors, glial cell derived‐neurotrophic factor and brain‐derived neurotrophic factor (BDNF), were not included in our differentiation medium while inducing ESCs toward neuronal fate. Using such simplified formula with reduced amount of components, it allowed us to further define the neurotoxic effect of ketamine in ESC‐derived neurons. Moreover, as we focused on investigating the functional mechanisms of miR‐107, and its downstream target genes, using defined medium and excluding exogenous growth factors seemed to be a necessary step to unravel the underlying molecular signaling pathway responsible for ketamine‐induced neural injury.

Second, our qRT‐PCR demonstrated that miR‐107 was upregulated by ketamine, in concentration‐dependent manner, in NSC‐derived neurons, suggesting that aberrant miR‐107 expression (upregulation) may be closely correlated with ketamine‐induced pathological conditions in brain. To support to such hypothesis, a previous publication demonstrated that miR‐107 was also upregulated in brain tissues induced by ischemic stroke 23. On the other hand, studies demonstrated that, in Alzheimer's disease, cortical miR‐107 was found to be aberrantly downregulated in both human patients and animal models 23, 24. Combining with the results in our study, it seems like brain‐injury induced miR‐107 deregulating pattern, either upregulation or downregulation, may very well be determined by the type of injury and (or) the location of injury in brain.

Also in our study, we revealed that miR‐107 downregulation could attenuate ketamine‐induced neural injury in NSC‐derived neurons. In pervious animal studies, researchers demonstrated that miRNAs could regulate ketamine‐induced neurotoxicity, such as neural apoptosis and memory impairment, in hippocampal CA1 or CA3 neurons 19, 20, 21. As compared to those studies, our study may bear much broader clinical implication as ESC‐derived neurons may represent more neuronal lineages than hippocampal neurons in the brain. Most importantly in our study, we discovered that BDNF was the direct downstream target of miR‐107 during the process of ketamine‐induced neural injury in NSC‐derived neurons. In a recent study, BDNF was suggested to be inversely correlated with long noncoding RNA, BDNF‐AS, in anesthesia‐injured neural stem cell‐derived neurons 29. However, no functional mechanism of BDNF was demonstrated in that study 29. Thus, our study is the first‐ever report to reveal a functional role of growth factor BDNF to co‐regulate with epigenetic factor (miR‐107) during the process of anesthesia‐induced neural injury in brain.

## CONFLICT OF INTEREST

None.
